# Multicenter phase II study on cisplatin, pemetrexed, and bevacizumab followed by maintenance with pemetrexed and bevacizumab for patients with advanced or recurrent nonsquamous non-small cell lung cancer: MAP study

**DOI:** 10.1186/s12885-018-5146-3

**Published:** 2018-12-10

**Authors:** Yasuhiro Tsutani, Yoshihiro Miyata, Takeshi Masuda, Kazunori Fujitaka, Mihoko Doi, Yoshikazu Awaya, Shoichi Kuyama, Soichi Kitaguchi, Kazuhiro Ueda, Noboru Hattori, Morihito Okada

**Affiliations:** 1 Department of Surgical Oncology, Research Institute for Radiation Biology and Medicine, Hiroshima University, 1-2-3 Kasumi, Minami-ku, Hiroshima, 734-8551 Japan; 2 Department of Respiratory Internal Medicine, Hiroshima University, 1-2-3 Kasumi, Minami-ku, Hiroshima, 734-8551 Japan; 3 Department of Medical Oncology, Hiroshima Prefectural Hospital, 1-5-54, Ujinakanda, Minami-ku, Hiroshima, 734-8530 Japan; 4 Department of Respiratory Medicine, Miyoshi Central Hospital, 10531 Higashisakaya, Miyoshi, 728-8502 Japan; 5 Department of Respiratory Medicine, Iwakuni Medical Center, 1-1-1 Atago, Iwakuni, 740-8510 Japan; 6 Department of Respiratory Medicine, Asa Citizens Hospital, 2-1-1 Kabeminami, Asakita-ku, Hiroshima, 731-0293 Japan; 7 Department of Thoracic Surgery, Yamaguchi University, 1-1-1 Minamikogushi, Ube, 755-8505 Japan

## Abstract

**Background:**

We evaluated the safety and efficacy of induction chemotherapy with bevacizumab followed by maintenance chemotherapy with bevacizumab for advanced non-small cell lung cancer (NSCLC) in this multicenter phase II study.

**Methods:**

Chemotherapy-naïve patient with stage IIIB–IV or recurrent nonsquamous NSCLC were eligible. We planned approximately four cycles of induction cisplatin (75 mg/m^2^), pemetrexed (500 mg/m^2^), and bevacizumab (15 mg/kg) followed by maintenance with pemetrexed (500 mg/m^2^) and bevacizumab (15 mg/kg) until disease progression. Progression-free survival (PFS) was the primary endpoint.

**Results:**

Forty patients received a median of four induction chemotherapy cycles. Of them, 35 (87.5%) patients received a median of nine maintenance chemotherapy cycles. The objective response was 70.6%, and the disease control rate was 97.1%. The median PFS was 10.8 (95% CI, 9.0–12.6), and overall survival was 48.0 (95% CI, 32.9–63.1) months. Median PFS of 23 patients with epidermal growth factor receptor (EGFR) mutations and of 16 patients without EGFR mutations were 12.9 (95% CI, 9.4–16.3) and 7.9 (95% CI, 1.1–14.7) months, respectively. Toxicities graded ≥3 included neutropenia (15%), anemia (15%), hypertension (7.5%), anorexia (7.5%), fatigue (7.5%), thromboembolic events (5%), jaw osteonecrosis (5%), nausea (2.5%), oral mucositis (2.5%), tumor pain (2.5%), hyponatremia (2.5%), and gastrointestinal perforation (2.5%). Treatment-related deaths were not found.

**Conclusions:**

In patients with advanced or recurrent nonsquamous NSCLC, induction chemotherapy with cisplatin, pemetrexed, and bevacizumab followed by maintenance chemotherapy with pemetrexed and bevacizumab is safe and effective regardless of their EGFR mutation status.

**Trial registration:**

UMIN Clinical Trial Registry: UMIN000005569. Registered date: May 8, 2011.

## Background

Vascular endothelial growth factor (VEGF) is a strong stimulator of endothelial cell proliferation. It is required for maintaining tumor vasculature in various tumor types [1, 2]. Bevacizumab is a recombinant humanized monoclonal antibody, and it inhibits binding of VEGF to its receptor [2]. Combination therapies of bevacizumab and platinum-based doublet chemotherapy followed by bevacizumab maintenance as a first-line treatment are more superior than platinum-based doublet chemotherapy with respect to the overall survival (OS) and progression-free survival (PFS) in patients with advanced nonsquamous non-small cell lung cancer (NSCLC) in randomized controlled trials, such as the Eastern Cooperative Oncology Group (ECOG) E4599 and AVAiL [3, 4]. A phase III trial that compared front-line cisplatin and gemcitabine with cisplatin and pemetrexed demonstrated a treatment-by-histology interaction, showing an improvement in OS with cisplatin and pemetrexed in patients with nonsquamous NSCLC [5]. Thereafter, in a large, randomized, phase III study, it was revealed that maintenance chemotherapy with pemetrexed was effective and was well-tolerated in patients with advanced nonsquamous NSCLC in whom the cancer did not show progression after induction chemotherapy with pemetrexed and cisplatin [6]. We hypothesized that induction therapy with cisplatin, pemetrexed, and bevacizumab followed by pemetrexed and bevacizumab maintenance would result in better survival in patients with advanced nonsquamous NSCLC. At the time of trial design in 2010, results of the AVAPERL [the randomized phase III study comparing the efficacy of maintenance therapy with bevacizumab (7.5 mg/kg) with that of maintenance therapy with bevacizumab (7.5 mg/kg) plus pemetrexed (500 mg/m^2^) after induction therapy with cisplatin (75 mg/m^2^), pemetrexed (500 mg/m^2^), and bevacizumab (7.5 mg/kg)] had not been reported [7].

In the present phase II trial, we aimed to evaluate the safety and efficacy of induction therapy with cisplatin (75 mg/m^2^), pemetrexed (500 mg/m^2^), and bevacizumab (15 mg/kg) followed by maintenance therapy with pemetrexed (500 mg/m^2^) and bevacizumab (15 mg/kg) in patients with advanced or recurrent nonsquamous NSCLC.

## Methods

### Eligibility criteria

Patients were required to have nonsquamous NSCLC and have not received any prior systemic chemotherapy, except preoperative/postoperative adjuvant chemotherapy or EGFR-tyrosine kinase inhibitor (TKI), with stage IIIB, stage IV, or recurrence disease after surgery; have measurable lesion that met the Response Evaluation Criteria in Solid Tumors (RECIST) version 1.1 [8]; be aged from 20 to 74 years; ECOG performance status of 0 or 1; and have adequate organ function within 1 week before study entry. The laboratory value requirements were as follows: hemoglobin level ≥ 9 g/dl, absolute neutrophil count ≥1500/mm^3^, platelet count ≥100,000/mm^3^, serum bilirubin levels < 1.5 mg/dl, serum aspartate aminotransferase and alanine aminotransferase levels < 100 IU/l, and serum creatinine levels < 1.5 mg/dl; have an estimated life expectancy of at least 90 days; and have signed the document of informed consent.

Patients were not eligible if they had metastases of the central nervous system or prior therapies for brain metastasis; had received radiotherapy for lung lesions; had a history of cardiac effusion that required treatment; had another active malignancy; had a history of hemoptysis or hemosputum; had a complication related to a bleeding episode, such as bleeding diathesis, an evidence of major thoracic blood vessel involvement, an evidence of cavity formation in the lung lesion, or an evidence of thrombosis; needed an anti-thrombosis drug during the study or were administered an anti-thrombosis drug within 10 days before enrollment; had a history of brain vascular disease with symptom, gastrointestinal perforation, diverticulitis, or fistula, symptomatic heart failure, unstable angina or arrhythmia that required treatment, cardiac infarction within 1 year before enrollment, any evidence of interstitial lung disease, superior vena cava syndrome, a cord compression, a serious non-healing wound or unhealed bone fracture, an uncontrollable ulcer, uncontrollable hypertension, or a serious concomitant active infection that needed antibiotics; had known sensitivity to any component of platinum or monoclonal antibody drugs; or pregnancy or lactation.

The current study (UMIN000005569) was planned on the basis of the Declaration of Helsinki and Good Clinical Practice guidelines and was approved by the institutional review boards of all participating hospitals. Signed informed consent forms were obtained from the patients.

### Treatment and evaluation

Prior to the administration of pemetrexed, all patients received folic acid and vitamin B12 supplementation and standard premedication with dexamethasone [2, 9, 10]. Patients received induction chemotherapy on day 1 of each 21-day cycle comprising cisplatin (75 mg/m^2^), pemetrexed (500 mg/m^2^), and bevacizumab (15 mg/kg). Induction chemotherapy was repeated every 3 weeks for a maximum of four cycles. After completion of at least three cycles of induction chemotherapy, patients underwent maintenance chemotherapy on day 1 of the 21-day cycle comprising pemetrexed (500 mg/m^2^) and bevacizumab (15 mg/kg). Every 3 weeks, maintenance chemotherapy was repeated until disease progression or intolerance.

Using the National Cancer Institute Common Toxicity Criteria version 4.0, patients were evaluated every 21 days to assess toxicity. Tumor response was assessed using computed tomography; it was performed every 6 weeks for 24 weeks and every 6 weeks thereafter until disease progression. The original RECIST criteria (version 1.1) were used to assess the response [8]. Central review of radiologic assessment was not performed.

### Statistical methods

PFS, defined as the time from enrollment to disease progression (as assessed by the investigator) or to death, was the primary endpoint of the study. OS, objective response rate, and toxicity were the secondary endpoints. A median PFS of at least 7 months compared with the historic rate of 4.8 months [5] would be considered as a favorable outcome. We estimated that 35 patients were needed to achieve an 80% power with a one-sided 0.1 level test in this single-stage, single-arm trial. An intent-to-treat approach was followed for analyzing all data. Kaplan–Meier method was used to calculate survival distributions. All statistical analyses were performed using the Statistical Package for the Social Sciences software (version 24.0; IBM, Armonk, NY, USA). A *p* value of < 0.05 was considered to be significant.

## Results

### Patients’ characteristics

We enrolled 40 eligible patients [median age, 64.5 years; 57.5% (23/40) men] to receive treatment between July 2011 and August 2014. Table 1 shows the patients’ characteristics. Of all patients, 95% (38/40) had adenocarcinoma, 75.0% (30/40) had stage IV disease, and 57.5% (23/40) had EGFR mutations.

**Table 1:** Patients’ characteristics

		*n* = 40
Age (range)		64.5 (34–73)
Gender	Male	23 (57.5%)
	Female	17 (42.5%)
Performance status	0	34 (85.0%)
	1	6 (15.0%)
Smoking history	Yes	23 (57.5%)
Histology	Adenocarcinoma	38 (95.0%)
	Pleomorphic carcinoma	1 (2.5%)
	Non-small cell lung cancer	1 (2.5%)
Stage	IIIB	1 (2.5%)
	IV	30 (75.0%)
	Postoperative recurrence	9 (22.5%)
EGFR mutation	Positive	23 (57.5%)
	Negative	16 (40.0%)
	Unknown	1 (2.5%)
Previous adjuvant chemotherapy	Yes	6 (15.0%)
Previous EGFR-TKI therapy	Yes	8 (20.0%)

*EGFR* epidermal growth factor receptor, *TKI* tyrosine kinase inhibitor

The 40 eligible patients were administered a median of four cycles (range, 1–4 cycles) of induction chemotherapy comprising cisplatin, pemetrexed, and bevacizumab. Of these patients, 35 (87.5%) were administered maintenance chemotherapy comprising pemetrexed and bevacizumab for a median of nine cycles (range, 1–54 cycles) (Table 2). Reasons for induction chemotherapy discontinuation were disease progression (*n* = 1), unacceptable toxicity (*n* = 3), and patient request (*n* = 1).

**Table 2:** Chemotherapy administration and objective response

Induction therapy (*n* = 40)	Median cycle (range)	4 (1–4)
Maintenance therapy (*n* = 35)	Median cycle (range)	9 (1–54)
Response (*n* = 34)	CR	0 (0%)
	PR	24 (70.6%)
	SD	9 (26.5%)
	PD	1 (2.9%)

### Toxicity

Table 3 shows the grade 3 or 4 treatment-related toxicities that developed in the patients. The proportion of patients developing toxicities graded > 3 included 15.0% with neutropenia, 15.0% with anemia, 7.5% with hypertension, 7.5% with anorexia, 7.5% with fatigue, 5% with a thromboembolic event, 5% with jaw osteonecrosis, 2.5% with nausea, 2.5% with oral mucositis, 2.5% with tumor pain, 2.5% with hyponatremia, and 2.5% with gastrointestinal perforation. There were no treatment-related deaths.

**Table 3:** Therapy toxicities (>Grade 3)

	Grade 3	Grade 4
Neutropenia	5 (12.5%)	1 (2.5%)
Anemia	6 (15.0%)	0 (0%)
Hypertension	3 (7.5%)	0 (0%)
Anorexia	3 (7.5%)	0 (0%)
Fatigue	3 (7.5%)	0 (0%)
Thromboembolic event	2 (5.0%)	0 (0%)
Jaw osteonecrosis ^a^	2 (5.0%)	0 (0%)
Nausea	1 (2.5%)	0 (0%)
Oral mucositis	1 (2.5%)	0 (0%)
Tumor pain	1 (2.5%)	0 (0%)
Hyponatremia	1 (2.5%)	0 (0%)
Gastrointestinal perforation	0 (0%)	1 (2.5%)

^a^These two patients received denosumab

### Efficacy outcomes

**Fig. 1:**
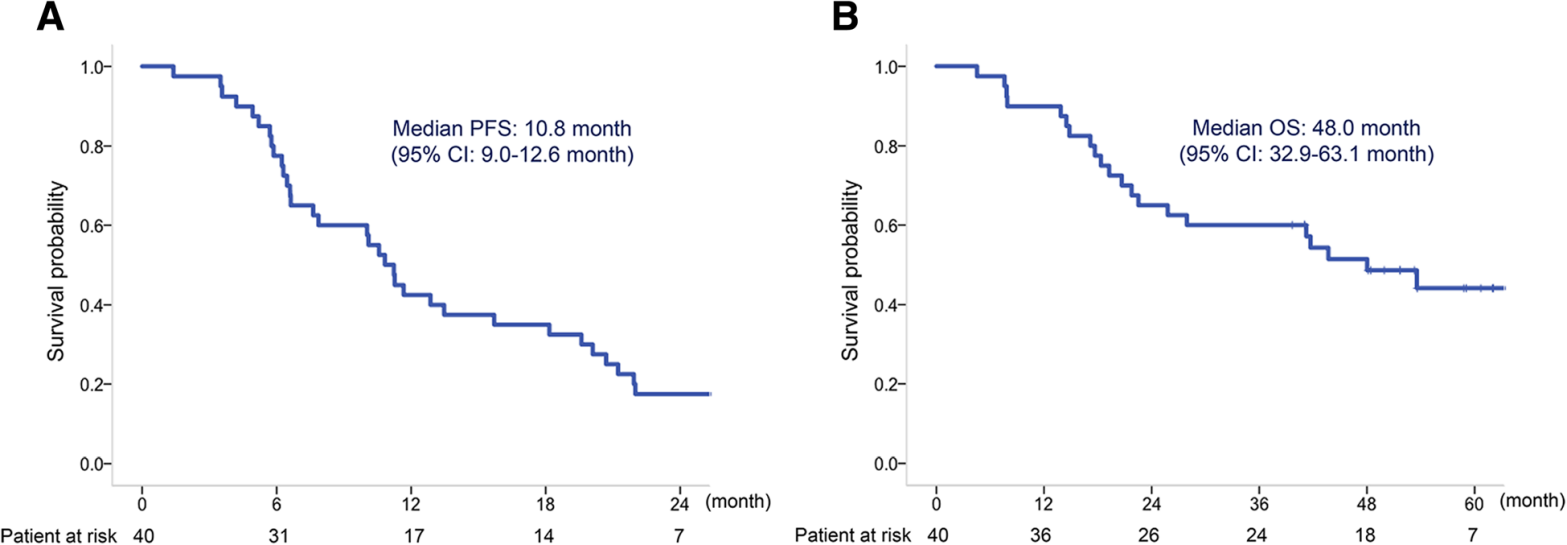
Progression-free survival (**a**) and overall survival (**b**) in patients with advanced or recurrent nonsquamous non-small cell lung cancer (*n* = 40)

**Fig. 2:**
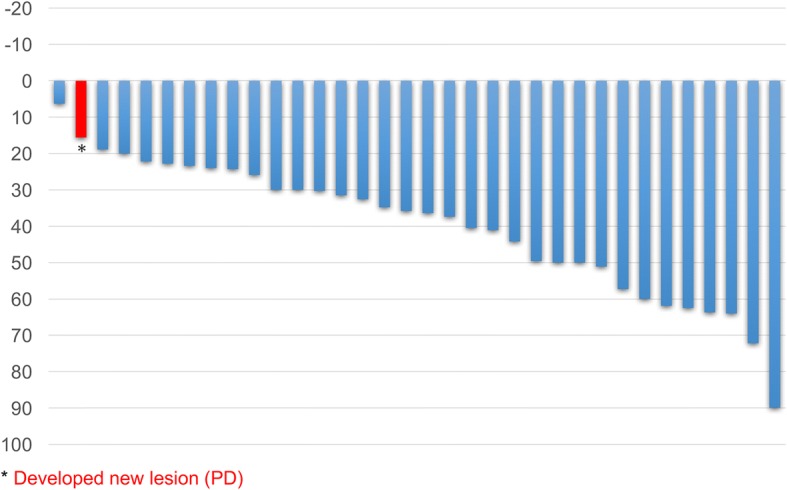
Best percentage change from the baseline in the sum of longest diameters of target lesions (*n* = 34)

This study met the primary endpoint. Median PFS was 10.8 (95% CI, 9.0–12.6), and OS was 48.0 (95% CI, 32.9–63.1) months (Fig. 1a and b). The objective response rate was 70.6%, and the disease control rate was 97.1% in patients with a measurable target lesion (*n* = 34) (Table 2). On analyzing the relative change in the tumor size from the baseline, we found that all patients (34/34) experienced some degree of tumor shrinkage (Fig. 2).

**Fig. 3:**
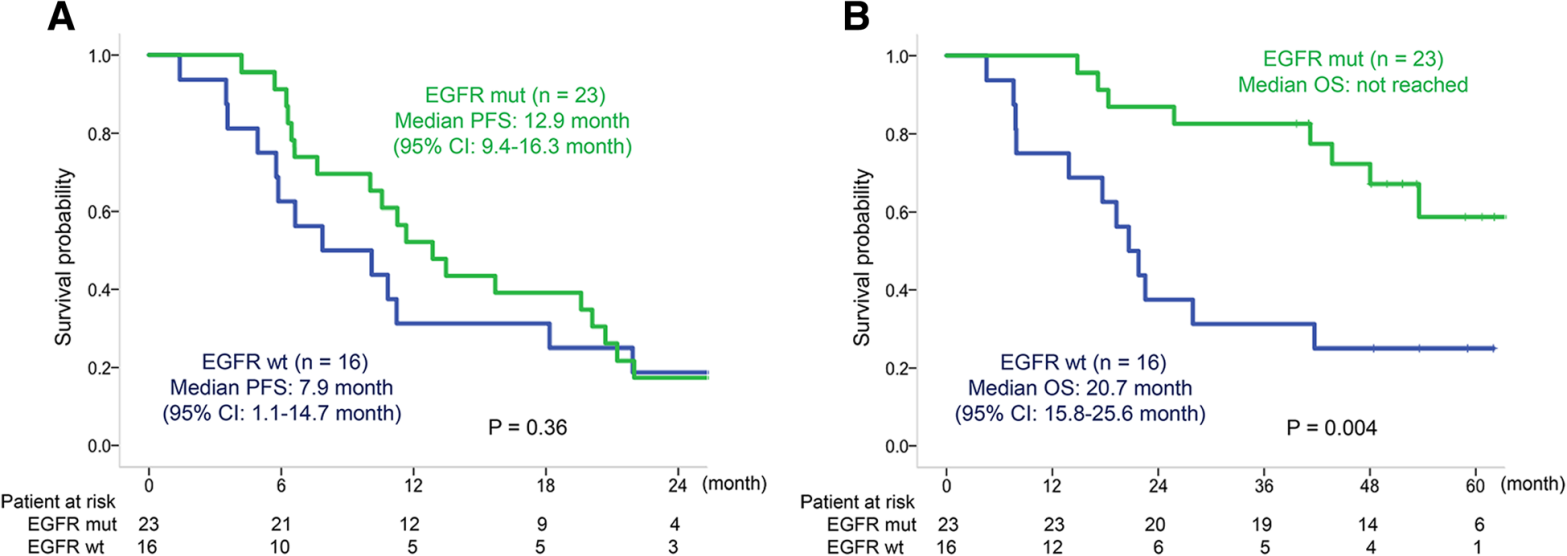
Progression-free survival (**a**) and overall survival (**b**) in patient subgroups based on their EGFR mutation status

The median PFS of patients with EGFR mutations (*n* = 23) and those without the mutation (*n* = 16) were 12.9 (95% CI, 9.4–16.3) and 7.9 (95% CI, 1.1–14.7) months, respectively (*P* = 0.36, Fig. 3a). Patients with EGFR mutations (n = 23) did not reach a median OS, and those without the mutation (*n* = 16) had a median OS of 20.7 (95% CI, 15.8–25.6) months (*P* = 0.004; Fig. 3b).

## Discussion

We demonstrated the safety and efficacy of induction therapy using cisplatin (75 mg/m^2^), pemetrexed (500 mg/m^2^), and bevacizumab (15 mg/kg) followed by maintenance therapy with pemetrexed (500 mg/m^2^) and bevacizumab (15 mg/kg) in patients with advanced or recurrent nonsquamous NSCLC. Non-hematological toxicities graded higher than 3 were not found in 10% of the patients, and a grade 4 non-hematological toxicity (gastrointestinal perforation) developed in only one (2.5%) patient. Furthermore, there was no treatment-related death in this study. The low frequencies of severe toxicities observed in this study are consistent with those observed in other studies [7, 11]. Serious adverse events such as pulmonary embolism (1.6%) and pneumonia (5.6%) occurred during maintenance therapy with pemetrexed and bevacizumab in the AVAPERL study, in which the bevacizumab dose was 7.5 mg/kg [7]. A retrospective study evaluating the feasibility of cisplatin, pemetrexed, and bevacizumab (15 mg/kg) in patients with advanced nonsquamous NSCLC reported low frequencies of grade 3/4 non-hematologic toxicities (hypertension, 13%; pulmonary thromboembolism, 3%; fatigue, 3%; anorexia, 6%; gastric ulcer, 3%; and colitis, 3%) and no treatment-related deaths [11]. To the best of our knowledge, this is the first prospective study evaluating 15 mg/kg of bevacizumab in combination with standard dose cisplatin and pemetrexed for patients with nonsquamous NSCLC; our results suggest that the dose of 15 mg/kg bevacizumab and combination therapy with cisplatin, pemetrexed, and bevacizumab followed by maintenance therapy with pemetrexed and bevacizumab is a safe and feasible regimen.

Regarding efficacy, this study met its primary endpoint with a median PFS of 10.8 months, which is consistent with the results of the phase III AVAPERL study (median PFS from induction treatment, 10.2 months) [7], and seems superior to those of the PointBreak study (carboplatin, pemetrexed, and bevacizumab followed by pemetrexed and bevacizumab: the median PFS from induction of treatment, 8.6 months) [9] and those of the Pronounce study (carboplatin plus pemetrexed followed by pemetrexed: the median PFS from induction of treatment, 4.44 months) [12] (Table 4). The objective response rate of 70.6% in the present study is also excellent.

**Table 4:** Progression-free survival and overall survival of cisplatin/carboplatin, pemetrexed with or without bevacizumab in patients with nonsquamous non-small cell lung cancer

Study	Phase	Induction therapy	Maintenance therapy	PFS (months)	OS (months)
Scagliotti, et al. [5]	3	CDDP + PEM	–	5.3	11.8
PARAMOUNT [6]	3	CDDP + PEM	PEM	6.9	–
AVAPEARL [7]	3	CDDP + PEM + BEV	PEM + BEV	10.2	NR
PointBreak [23]	3	CBDCA + PEM + BEV	PEM + BEV	8.6	17.7
PRONOUNCE [12]	3	CBDCA + PEM	PEM	4.44	10.5
MAP (The current study)	2	CDDP + PEM + BEV	PEM + BEV	10.8	48.0

*PFS* progression-free survival, *OS* overall survival, *CDDP* cisplatin, *PEM* pemetrexed, *BEV* bevacizumab, *CBDCA* carboplatin, *NR* not reached

This study included 57.5% patients with EGFR mutations. In our EGFR status-based sub-analysis, PFS of patients with EGFR mutations seemed longer (median PFS, 12.9 months) than that of those without the mutations (median PFS, 7.9 months); however, this difference was not significant. PFS of patients with EGFR mutations who received cisplatin, pemetrexed, and bevacizumab in the present study was similar to that of those who received EGFR-TKIs in other studies (gefitinib, PFS of 9.2–10.8 months; erlotinib, PFS of 9.7–13.1 months; and afatinib, PFS of 11–11.1 months) [13–18]. The standard first-line treatment of patients with advanced NSCLC and with EGFR mutation usually includes EGFR-TKIs; however, a large retrospective study in a real world clinical setting suggested that in patients with NSCLC and EGFR mutations, sequential use of chemotherapy in addition to EGFR-TKI improved OS compared with the use of EGFR-TKI alone [19]. It is important not to miss the opportunity of using the most effective chemotherapy in such cases. Combination therapy with EFGR-TKI and cytotoxic agents may be another way to avoid missing to use chemotherapy, as a phase II study suggested promising results of concurrent use of gefitinib, carboplatin, and pemetrexed [20]. Additional use of anti-angiogenic treatment is also promising for patients with NSCLC with EGFR mutations. In addition to results of the current study, a randomized phase II study reported that erlotinib with bevacizumab showed better PFS than erlotinib alone [21]. Moreover, a recent phase III study demonstrated that combination therapy with atezolizumab, bevacizumab, carboplatin, and paclitaxel significantly improved PFS and OS compared with bevacizumab, carboplatin, and paclitaxel even in patients with EGFR mutations [22]. Chemotherapy and bevacizumab as well as EGFR-TKI may be potential key drugs to improve prognosis of patients with nonsquamous NSCLC with EGFR mutations. The excellent OS of patients with EGFR mutations in this study, with over 50% patients with a 5-year OS rate, indicates that induction therapy with cisplatin, pemetrexed, and bevacizumab followed by maintenance therapy with pemetrexed and bevacizumab can be an optional first-line treatment for patients with advanced nonsquamous NSCLC and with EGFR mutations.

## Conclusions

A first-line combination therapy with cisplatin (75 mg/m^2^), pemetrexed (500 mg/m^2^), and bevacizumab (15 mg/kg) followed by maintenance with pemetrexed (500 mg/m^2^) and bevacizumab (15 mg/kg) is safe and effective for patients with stage III, IV, or recurrent nonsquamous NSCLC, and even for those with EGFR mutations.

## Abbreviations

ECOG: Eastern Cooperative Oncology Group
EGFR: Epidermal growth factor receptor
NSCLC: Non-small cell lung cancer
OS: Overall survival
PFS: Progression-free survival
TKI: Tyrosine kinase inhibitor
VEGF: Vascular endothelial growth factor
